# Discovery of an 8-oxoguanine regulator PCBP1 inhibitor by virtual screening and its synergistic effects with ROS-modulating agents in pancreatic cancer

**DOI:** 10.3389/fmolb.2024.1441550

**Published:** 2024-08-07

**Authors:** Kexiong Qiao, Chengjie Xu, Chaolei Zhang, Qianqian Wang, Jun Jiang, Zongrong Chen, Liangjing Zhou, Shengnan Jia, Liping Cao

**Affiliations:** ^1^ Department of General Surgery, Sir Run Run Shaw Hospital, Zhejiang University School of Medicine, Hangzhou, Zhejiang Province, China; ^2^ Department of Emergency Medicine, Sir Run Run Shaw Hospital, Hangzhou, Zhejiang Province, China; ^3^ School of Medicine, Sir Run Run Shaw Hospital, Graduate School, Zhejiang University, Hangzhou, Zhejiang, China; ^4^ Zhejiang Engineering Research Center of Cognitive Healthcare, Sir Run Run Shaw Hospital, Zhejiang University School of Medicine,, Hangzhou, Zhejiang Province, China

**Keywords:** poly (rC)-binding protein 1 (PCBP1), virtual screening, molecular dynamics simulation, silychristin, R-loop, pancreatic cancer

## Abstract

**Introduction:** Drugs that target reactive oxygen species (ROS) metabolism have progressed the treatment of pancreatic cancer treatment, yet their efficacy remains poor because of the adaptation of cancer cells to high concentration of ROS. Cells cope with ROS by recognizing 8-oxoguanine residues and processing severely oxidized RNA, which make it feasible to improve the efficacy of ROS-modulating drugs in pancreatic cancer by targeting 8-oxoguanine regulators.

**Methods:** Poly(rC)-binding protein 1 (PCBP1) was identified as a potential oncogene in pancreatic cancer through datasets of The Cancer Genome Atlas (TCGA) project and Gene Expression Omnibus (GEO). High-throughput virtual screening was used to screen out potential inhibitors for PCBP1. Computational molecular dynamics simulations was used to verify the stable interaction between the two compounds and PCBP1 and their structure–activity relationships. *In vitro* experiments were performed for functional validation of silychristin.

**Results:** In this study, we identified PCBP1 as a potential oncogene in pancreatic cancer. By applying high-throughput virtual screening, we identified Compound 102 and Compound 934 (silychristin) as potential PCBP1 inhibitors. Computational molecular dynamics simulations and virtual alanine mutagenesis verified the structure–activity correlation between PCBP1 and the two identified compounds. These two compounds interfere with the PCBP1–RNA interaction and impair the ability of PCBP1 to process RNA, leading to intracellular R loop accumulation. Compound 934 synergized with ROS agent hydrogen peroxide to strongly improve induced cell death in pancreatic cancer cells.

**Discussion:** Our results provide valuable insights into the development of drugs that target PCBP1 and identified promising synergistic agents for ROS-modulating drugs in pancreatic cancer.

## 1 Introduction

Pancreatic cancer is the fourth leading cause of cancer deaths worldwide (Yang and Kimmelman, 2011), yet limited progress towards effective targets has been made. The desmoplastic reaction and dense stroma of pancreatic cancer make a high lack of nutrients and metabolic reprograming unique features of pancreatic cancer, which generates high concentrations of reactive oxygen species (ROS) (Yang et al., 2019). Despite numerous attempts to treat pancreatic cancer by regulating ROS levels with, for example, gemcitabine, cisplatin, or erastin, the efficacy remains poor because of the adaptation of cancer cells to high ROS concentrations (Abdel Hadi et al., 2021). Therefore, it is vital to inhibit antioxidant systems of pancreatic cancer for synergistic effect with existing chemotherapeutic drugs.

Biomolecules such as lipids, proteins, and nucleic acids undergo oxidative modifications in response to ROS, which leads to mitochondrial dysfunction and activate cell death pathways (Yarana et al., 2018). 8-Oxoguanine (o8G) is the most prevalent form of oxidative nucleic acid modification because the guanine base has the lowest redox potential (Hahm et al., 2022). o8G in RNA is the most abundant epitranscriptional modification in oxidized cells (Hahm et al., 2022). As a result of oxidative damage, o8G is directly written by ROS and read by RNA-binding proteins (YB-1, AUF1, PCBP1, and PCBP2) and ribonucleolytic enzymes (PNPase and APE1) (Hahm et al., 2022), which initiates various cell fates, including carcinogenesis or activation of RNA decay machinery (Shan et al., 2003; Chang et al., 2008; Vascotto et al., 2009; Liu et al., 2012; Simms et al., 2014; Seok et al., 2020). Eom et al. showed that o8G modification of microRNA reprogrammed by redox changes contributed to malignancy of hepatocellular carcinoma (Eom et al., 2023). By binding to o8G residues, AUF1 recognized oxidatively damaged RNA and initiated selective mRNA decay, which eliminated incorrectly formed RNA and protected organisms from oxidative damage (Ishii and Sekiguchi, 2019). Therefore, o8G regulatory factors may be promising antioxidant targets in pancreatic cancer, but little is known about the o8G regulating pathway or the potential drug targets.

Poly (rC) binding proteins (PCBPs) belong to the heterogeneous nuclear ribonucleoprotein (hnRNP) family and are characterized by triple K homology (KH) domains and poly (rC) binding specificity (Yanatori et al., 2020). Among the PCBPs, only PCBP1 and PCBP2 were found to respond to oxidative conditions by binding heavily oxidized RNA (Ishii et al., 2020). PCBP1, which is considered to be a tumor suppressor in various types of cancer, regulates gene expression in multiple ways, including transcription, alternative splicing, and translation. Besides its role in adaptation to oxidative stress, PCBP1 also functions as an iron chaperone to regulate the iron storage pathway (Yanatori et al., 2020). However, molecules that target PCBP1 remain largely unexplored.

In this study, we identified PCBP1 as a potential oncogene in pancreatic cancer. We performed structure-based virtual screening and found two small molecule inhibitors, Compound 102 and Compound 934, that specifically targeted the RNA binding KH domain of PCBP1, but not that of PCBP2. Molecular dynamics simulations confirmed a stable interaction between the two compounds and PCBP1 and their structure–activity relationships. Moreover, we found that these two compounds interfered with the PCBP1–RNA interaction, and impaired the ability of PCBP1 to process RNA, leading to R loop accumulation. Compound 934 (silychristin) strongly enhanced the ROS agent hydrogen peroxide to induce cell death in pancreatic cancer cells. Our results provide valuable insights into the development of drugs that target PCBP1, which showed promising synergistic effects with ROS-modulating drugs in pancreatic cancer.

## 2 Materials and methods

### 2.1 Cell culture

Human pancreatic cancer cell lines PANC-1 was purchased from the American Type Culture Collection (ATCC) and cultured in DMEM (Corning, United States, 27920011) supplemented with 10% fetal bovine serum (Cellmax, China, SA211.02), 1% penicillin–streptomycin (Biosharp, China, BL505A) at 37°C in a humid incubator with 5% CO2. All the cells used in this study were routinely tested for mycoplasma contamination.

### 2.2 Cell viability assay and reagents

Pancreatic ductal adenocarcinoma (PDAC) cells were cultured with the indicated treatment in a 96-well plate and incubated in new culture medium with cell counting kit 8 (CCK-8) solution according to the manufacturer’s instructions (Abbkine, China, ATWF08021). Optical density values at 450 nm were measured using a microplate reader (Thermo Fisher Scientific, Madrid, Spain). The following reagents were used in the assay: hydrogen peroxide solution (Hengjian, China) and silychristin (Compound 934 in this study) (Solarbio, China, IS3560).

### 2.3 Immunofluorescence staining

Cells grown in chamber slides were washed with phosphate-buffered saline (PBS) and fixed by ice-cold methanol for 15 min at 4°C. After permeabilization by 0.1% Triton X-100 for 5 min on ice, the cells were blocked by 5% bovine serum albumin dissolved in PBS. Then, the slides were incubated in anti-S9.6 (Kerafast, ENH001, 1:200) primary antibody for 1 h and secondary antibody (Thermo Fisher Scientific, 1820027 for anti-mouse and 1820538 for anti-rabbit, 1:2,000) for 20 min at room temperature. After washing by PBS three times, the slides were mounted using mounting medium with DAPI (Abcam, United Kingdom, ab104139) and stored at 4°C. The fluorescence images were acquired using a confocal microscope Nikon A1 Ti (Japan). All procedures after primary antibody incubation were performed in the dark.

### 2.4 Data acquisition and bioinformatic analysis

Sequence and three-dimensional structure data of PCBP1 and PCBP2 were downloaded from the National Centre for Biotechnology Information (NCBI) and the Protein Data Bank (PDB). RNA sequencing and clinical information of the TCGA-PAAD cohort were obtained from The Cancer Genome Atlas Program data portal (https://portal.gdc.cancer.gov/). Gene expression data for normal and pancreatic cancer tissues were downloaded from the Gene Expression Omnibus (GEO: GSE196009). All the datasets used in this study are publicly available. Analysis of PCBP1 and PCBP2 expression in cancer and normal tissues and overall survival analysis were performed using GEPIA 2.0 (Tang et al., 2019).

### 2.5 Protein and ligand preparation

We used the protein preparation wizard of the Schrödinger suite (Sastry et al., 2013) to prepare the PCBP–ligand complex as follows: 1) add missing hydrogen atoms; 2) correct metal ionization states; 3) enumerate bond orders in HET groups in the PDB file; 4) determine ligand protonation states and associated energy penalties; 5) optimize protonation states of histidine residues and the protein hydrogen bond network; 6) rectify potentially transposed heavy atoms; and 7) perform a restrained minimization. All the natural compound structures sourced from the TargetMol natural compound library were prepared by the LigPrep module (Greenwood et al., 2010). The pH range for this module was set as 7.0 ± 2.0. The OPLS3e force field was used for structural validation and energy minimization (Roos et al., 2019). The binding region of the pyrimidine derivative was identified as the target site, and a corresponding grid was created.

### 2.6 Structure-based virtual screening and *in silico* absorption, distribution, metabolism, and excretion (ADME) analysis

We performed structure-based virtual screening by applying the Glide algorithms to identify hit compounds against PCBP1 and PCBP2 (Friesner et al., 2004). The chemical compounds database was Docking Zinc in-man subset (https://zinc.docking.org/substances/subsets/in-man/). High-throughput virtual screening, standard precision (SP), and extra precision (XP) were included. Briefly, the top 10% of the high-throughput virtual screening results were selected and transferred to Glide SP. The top 10% of the SP results were retained for Glide XP, and the top 10% of XP results were determined. The Prime Molecular Mechanics-Generalized Born Model and Solvent Accessibility (MM-GBSA) (Hou et al., 2011) values of the indole derivative compounds were calculated for ranking. In silico ADME was performed using ADMETlab 2.0 (Xiong et al., 2021). The cut-off value was set referring to our published work (Guo et al., 2023).

### 2.7 Binding pose metadynamics

Before the metadynamics simulations, the system was prepared in a simple point-charge (SPC) water box. After energy minimization and constraint application, the temperature was increased gradually to 300 K. The last 0.5 ns of unbiased molecular dynamics (MD) simulation served as the reference for the subsequent metadynamics protocol. Hill height of 0.05 kcal/mol and width of 0.02 Å were used for the metadynamics simulations. Root mean square deviation (RMSD) was calculated by applying a distance of 3 Å between protein residues and ligands. PoseScore, PersScore, and CompScore were calculated to assess the stability of ligand binding. PoseScore (average RMSD from the ligand’s initial pose) <2 Å was considered stable for the protein–ligand complex (Fusani et al., 2020). PersScore calculated hydrogen bonds during the simulations. A high PersScore indicates high stability. CompScore was calculated by linearly combining PoseScore and PersScore (Jin et al., 2023). A low CompScore indicates high stability.

### 2.8 Molecular dynamics (MD) simulation

We performed all-atom MD simulations using the Desmond module of the Schrödinger suite in the initial phase. The simulations were performed within Maestro, starting with docked complexes that were placed in a cubic water box with buffer distance of 10 Å. The SPC water model and 0.15 M NaCl were introduced for physiological relevance. The particle-mesh Ewald method was used for long-range electrostatic interactions (Simmonett and Brooks, 2021); short-range van der Waals and Coulomb interactions were cut off at 9.0 Å. After solvation, the systems underwent minimization and equilibration using the default Desmond protocol in Maestro, which includes involved restrained simulations in both the NVT (constant number of particles, volume, and temperature) and NPT (constant number of particles, pressure, and temperature) ensembles. A 100 ns MD simulation was performed in the NPT ensemble with periodic boundary conditions after equilibration. The OPLS4 force field was used to describe interatomic interactions. The temperature was maintained at 300 K using the Nosè-Hoover chain thermostat, and the pressure was kept at 1 atm using the Martyna-Tobias-Klein barostat method. Subsequent to the initial MD simulations, a multi-step simulation protocol was followed. This protocol included Brownian dynamics simulations in the NVT ensemble at 10 K, with restraints applied on solute heavy atoms for 100 ps. Additional stages involved NVT simulations at 10 K with small timesteps and restraints on solute heavy atoms for 12 ps each. A subsequent NPT simulation at 10 K with restraints on solute heavy atoms was performed for 12 ps, followed by NPT simulations with and without restraints, both lasting 12 ps. Finally, a long NPT simulation of 500 ns was carried out, maintaining a pressure of 1 atm using the Martyna-Tobias-Klein barostat method and a temperature of 300 K using the Nosè-Hoover chain thermostat. MD simulations in the intracellular background were conducted under 0.15 M KCl concentration. *In vivo* simulations were conducted with intracellular conditions and at 310 K.

### 2.9 siRNA silencing, RNA extraction and quantitative reverse transcription (RT-qPCR) assay

siPCBP1 were purchased from RiboBio (Guangzhou, China) and transfected into PANC-1 cells with GenMute siRNA Transfection Reagent (SignaGen, United States, SL100568) following the manufacturer’s instructions. The siRNA sequences are as follows: siPCBP1-1: 5′-CGGGTGTAAGATCAAAGAGAT-3′; siPCBP1-2: 5′- GCCTACTCGATTCAAGGACAA-3′; siPCBP1-3: 5′- GCCATCTTTAAGGCTTTCGCT-3′. For RNA extraction, total RNA was isolated by Ultrapure RNA Kit (Cwbio, China, CW0581) and quantified by NanoDrop spectrophotometer (Thermo, United States). The RNA was reverse transcribed to cDNA (gDNA cleared) by Hifair ® Ⅱ 1st Strand cDNA Synthesis Kit (Yeason, China, 11121ES60). The cDNA was further used in real time PCR by Hieff UNICON ® qPCR SYBR Green Master Mix (Yeason, China, 11200ES03) and ABI Q6 real-time PCR System (Applied Biosystems, Thermo Fisher Scientific, United States). The expression level of PCBP1 was analysed using the 2^−ΔΔCt^ method with normalization of Actin mRNA. Primer sequences are as follows: Actin-F: 5′- CCAACCGCGAGAAGATGAC-3′; Actin-R: 5′- GAGTCCATCACGATGCCAGT-3′; PCBP1-F: 5′-AAAGGCGGGTGTAAGATCAAAG-3′; PCBP1-R: 5′-GGCAAATCTGCTTGACACACTC-3′

### 2.10 Statistical analysis of the data

The data were analyzed using GraphPad Prism 8.0 (GraphPad Prism software, United States). Each experiment was performed at least in triplicate unless otherwise stated. Student’s t-test, Kruskal-Wallis test, Kaplan-Meier method, and Wilcoxon rank sum test were applied. *P* < 0.05 was considered to be statistically significant.

## 3 Results

### 3.1 High expression of PCBP1 predicts poor prognosis in pancreatic cancer

**FIGURE 1:**
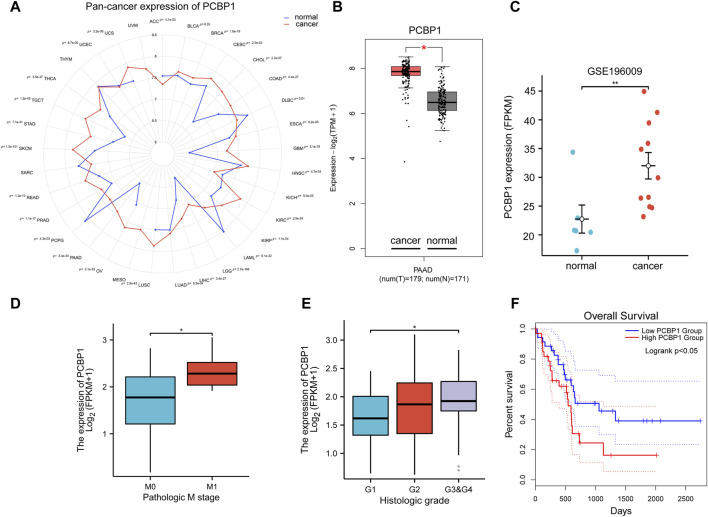
High expression of PCBP1 predicts poor prognosis in pancreatic cancer **(A)** Pan-cancer expression of PCBP1 in TCGA and GTEx datasets. **(B)** PCBP1 expression in TCGA-PAAD project and GTEx datasets (cancer vs. normal, *P* = 3.4e-34). **(C)** PCBP1 expression in GSE196009 cohort (normal vs. cancer, *P* = 0.0048). **(D)** PCBP1 expression in different metastasis stage groups of TCGA-PAAD (M0 vs. M1, *P* = 0.0296). **(E)** PCBP1 expression in different histologic grade groups of TCGA-PAAD (G1 vs. G3&G4, *P* = 0.03). **(F)** Overall survival of low/high expression of PCBP1, 20% and 80% are cut-off value for low or high expression group respectively (logrank *P* = 0.049).

Pan-cancer expression data from The Cancer Genome Atlas Program (TCGA) and GTEx cohorts database showed that the expression of PCBP1 was significantly higher in most types of cancer compared with its expression in normal tissues (23/33) (Figure 1A), including pancreatic cancer (Figure 1B). The expression data from the GSE196009 cohorts further validated the high expression of PCBP1 in pancreatic cancer (Figure 1C). By applying clinical information in the TCGA-PAAD program, we found that PCBP1 expression was higher in patients with distant metastasis (Figure 1D). The histological grading stage showed that the expression of PCBP1 was much higher in poorly differentiated tumors (G3/4) compared with its expression in well differentiated cases (G1) (Figure 1E). Sequential Kaplan-Meier survival analysis based on dichotomy, tertile, quartile and quintile were performed. All of the low-expression groups showed a better prognosis and difference became significant gradually. (Figure 1F; Supplementary Figure S1). These results strongly suggest that PCBP1 may be a potential drug target in pancreatic cancer.

### 3.2 High-throughput virtual screening identifies compound 102 and compound 934 as potential PCBP1 inhibitors

**FIGURE 2:**
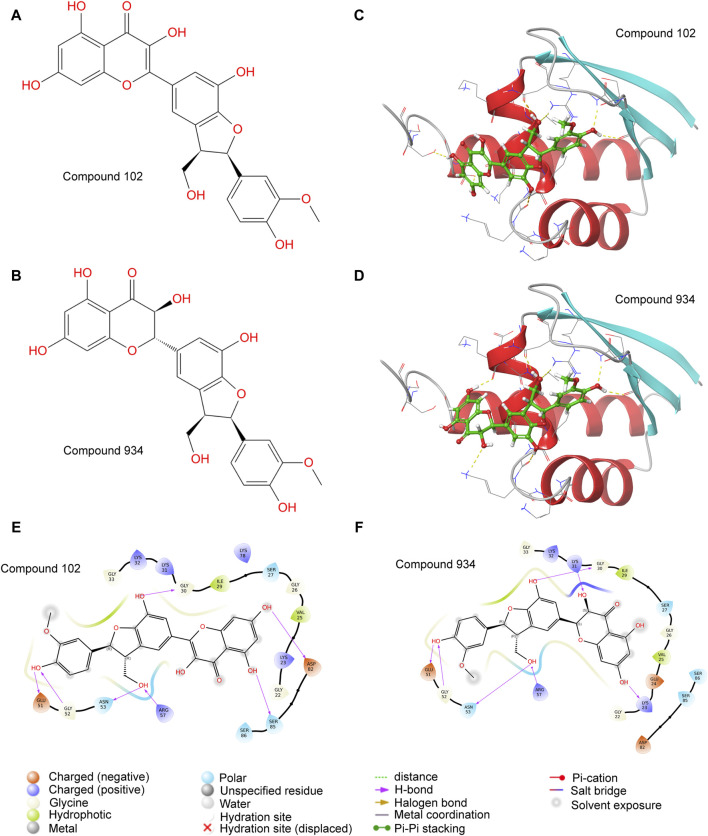
Molecular docking identifies Compound 102 and 934 as potential PCBP1 inhibitors **(A,B)** molecular structure of Compound 102 and 934. **(C,D)** molecular docking of Compound 102 and 934 binding to PCBP1. **(E,F)** Binding pattern diagrams of Compound 102 and 934.

We chose a library of compounds from Docking Zinc which had already been used in human for the purpose of drug safety. Whether the drug was commercialized or easy to synthesize was another concern when choosing the compounds library. Since the analyzed structure of PCBP1 did not contain the disordered domain, we believed that this part of the structure could be easily degraded. So, we selected the stabilized structure of PCBP1 that had already been analyzed (PDB ID: 3VKE) and discarded the structure containing disordered domain predicted by AlphaFold 2. The virtual screening targeting PCBP1, successfully rearranged the docking outcomes via MM-GBSA evaluations (Supplementary Table S1). Only Compound 102 and Compound 934 had significant affinity towards PCBP1 (Figures 2A, B), with energy levels for the complex below −40 kcal/mol. For Compound 934, the docking evaluation was −6.500 and the MM-GBSA binding free energy was −49.19 kcal/mol. For Compound 102, the docking evaluation was −6.007 and the MM-GBSA binding free energy was −42.96 kcal/mol. Compound 934 had a state penalty of 0.0161 kcal/mol and ligand strain energy of 3.881 kcal/mol, whereas Compound 102 had a state penalty of 0.0358 kcal/mol and ligand strain energy of 6.87 kcal/mol.

PCBP2, another member of the hnRNPE family (Yanatori et al., 2020), shares highly homology with PCBP1 in sequence and structure, but it was not correlated with prognosis in pancreatic cancer (Supplementary Figures S2A–E). We also docked molecules to PCBP2 to evaluate the specificity of the two compounds. The results are shown in Supplementary Table S2. Compared with their affinity for PCBP1, Compound 934 and Compound 102 showed no significant affinity for PCBP2; the docking evaluation values were only −3.289 and −2.787, respectively. In order to search for possible mutants of PCBP1 in pancreatic cancer, we used mutations and CNA data of TCGA-PAAD project in the cbioportal database (https://www.cbioportal.org/). Results showed that the percentage of TCGA pancreatic cancer patients harboring PCBP1 mutations was less than 1%. Therefore, we believed that PCBP1 variants were not in consideration for the virtual screening (Supplementary Figure S2F).

Visualization of the protein–ligand complexes showed that Compound 102 formed hydrogen bonds or salt bridges with the Gly-30, Glu-51, Gly-52, Asn-53, Arg-57, Asp-82, and Ser-85 residues of PCBP1 (Figures 2C, E). Compound 934 also formed hydrogen bonds or salt bridges with the Gly-30, Glu-51, Gly-52, Asn-53, and Arg-57 residues of PCBP1, but differed from Compound 102 by forming hydrogen bonds with the Lys-23 and Lys-31, including an additional double bond (Figures 2D, F).

Although the key residues we identified in the interaction between Compound 102 or 934 and PCBP1 are highly conserved in PCBP2 in sequence (Supplementary Figure S1A), the spatial conformations near the predicted binding sites are different, which ensure the drug specificity between these two proteins. Compound 102 and 934 were docked to PCBP1(magenta) or PCBP2 (blue). The protein-ligand complexes were merged to show different spatial conformations near the binding sites (Supplementary Figure S3).

The ADME properties of molecules are pivotal for drug development. The ADME pharmacokinetic parameters of Compound 934 and Compound 102 are shown in Supplementary Table S3. Compound 102 had a moderate partition coefficient and lipid–water partition coefficient, implying good cell membrane permeability. However, its low solubility and significant P-glycoprotein inhibition limited its absorption and excretion processes. Compound 934 had lower P-glycoprotein inhibition than Compound 102, implying less risk of drug–drug interactions. However, its high clearance means that the drug stays in the body for a shorter time and may require more frequent dosing.

In summary, the virtual screening and MM-GBSA scoring analysis highlighted Compound 102 and Compound 934 as potential PCBP1 inhibitors, with strong affinity and interaction dynamics, as well as better specificity for PCBP1 than for PCBP2. These findings confirm their viability as leading compounds for the advancement of innovative therapeutic agents aimed at PCBP1.

### 3.3 MD simulations of interactions between the two compounds and PCBP1

**FIGURE 3:**
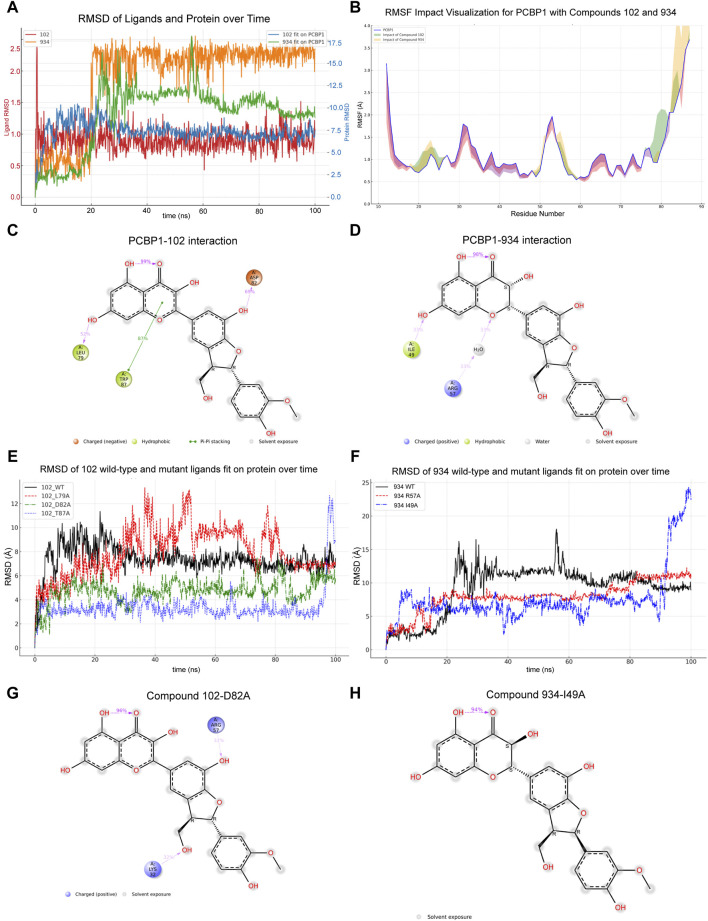
Molecular dynamic simulations of interaction between compounds and PCBP1 **(A)** Dynamic changes of RMSD for compounds and PCBP1 over time **(B)** Distribution of RMSF for different residues **(C,D)** Schematic diagram showing the detailed atomic interactions of Compound 102 and 934 with PCBP1. **(E,F)** Time-dependent RMSD of Compounds Fit on wild-type or mutant PCBP1 Complexes. **(G,H)** Detailed protein and ligand interactions after alanine mutagenesis of PCBP1.

Virtual screening offers only a snapshot of potential molecular interactions. To determine the interactions between the compounds and PCBP1 over time, we performed MD simulations *in vitro*, each extending over 100 ns. We used the simulation results to assess the stability of the binding, identify affected regions, and formulate a model that correlates structure and activity based on statistical analysis of interactions. First, we tracked the changes in the RMSD for both compounds and assessed their alignment with PCBP1 over the time period (Figure 3A). The complex with Compound 102 had outstanding stability because a stable interaction conformation was rapidly established. Conversely, the complex with Compound 934 in the PCBP1 binding pocket only began to show signs of stabilization after approximately 20 ns, then maintained a consistent binding conformation without notable alterations until the 85 ns mark.

The impact of the compounds on PCBP1 was assessed by measuring the root mean square fluctuation (RMSF) pre- and post-binding. The results show that both compounds had a similar influence by on protein flexibility (Figure 3B), namely reduced flexibility in positions 12–18, 28–47, and 60–77, and increased flexibility in positions 19–27, 48–59, and 78–87, largely acting to confine the flexibility of PCBP1.

Having established that both compounds can form a stable complex with PCBP1 and effectively limit its flexibility, we further dissected the molecular interactions observed during the simulations. Dynamic changes in the contacts between the compounds and PCBP1 residues over time are shown in Supplementary Figures S4A, B. Residues with interaction frequencies >30% are depicted in Figures 3C, D. The principal interaction sites between Compound 102 and PCBP1 were Leu-79, Asp-82, and Trp-87, and between Compound 934 and PCBP1 they were Ile-49 and Arg-57.

We conducted alanine mutagenesis to verify the structure–activity correlation between PCBP1 and the two compounds. The RMSD values idicated that the mutated residues were less able to form stable interactions with the molecules than the wild type residues were (Figures 3E, F). D82A and I49A mutations of PCBP1 residues led to significant changes in the binding of the two compounds near the interaction site as highlighted in the atomic interactions and torsion angle radar plots (Figures 3G, H; Supplementary Figures S4C, D).

Most proteins are post-translationally modified before being anchored in the membrane. The common form of post-translational modification for PCBP1 is phosphorylation at Ser43 (Brown et al., 2016). We conducted MD simulation after introducing artificial phosphorylation at Ser43. The RMSD values indicated that only Compound 934 was able to form stable interactions with phosphorylated PCBP1(Supplementary Figure S5).

### 3.4 Compound 102 and compound 934 interfere with the PCBP1–RNA interaction

**FIGURE 4:**
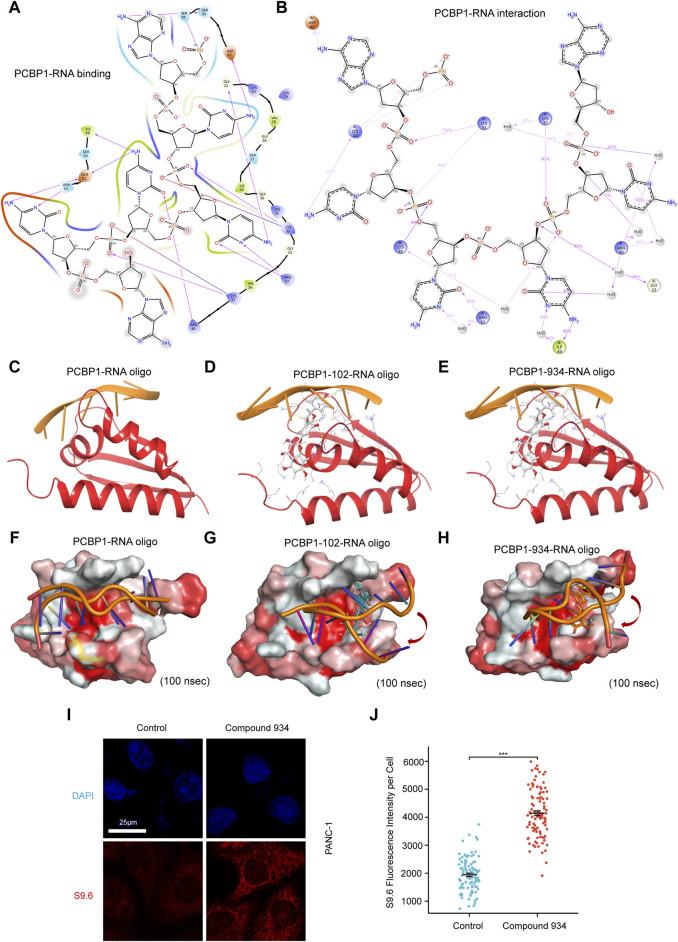
Compound 102 and 934 interfere PCBP1-RNA interaction **(A)** Binding pattern diagrams of nucleic acid and PCBP1 **(B)** Schematic diagram showing the detailed atomic interactions of RNA with PCBP1 **(C–E)** Docking exercises of RNA and PCBP1 after introduction of Compound 102 and 934. **(F–H)** 100 ns molecular dynamics simulation of RNA and PCBP1 after introduction of Compound 102 and 934. Surface of PCBP1 was colored by color_h.py. White is hydrophilic area, while red is hydrophobic area. **(I)** Detection of R-loop by S9.6 antibody immunofluorescence in PDAC cells treated by Compound 934 (silychristin, 80 μM, 24 h) **(J)** Quantification of **(I)**: the immunofluorescence intensity of S9.6 per cell (n > 100 cells) (*P* = 4.72e-50).

PCBP1 functions by binding or processing RNA. In the previous docking study, we noted that the Glu-51, Asn-53, Arg-57, Asp-82, and Ser-85 interaction sites overlapped with documented PCBP1 nucleic acid binding sites (Yoga et al., 2012). The binding pattern between PCBP1 and a nucleic acid is shown in Figure 4A. The molecular interactions observed during the simulations showed that the principal PCBP1–RNA interaction sites included Lys-23, Lys-31, Lys-32, Gys-33, Lys-37, Arg-40, Ile-49, Arg-57, and Asp-82 (Figure 4B). Compound 102 shared an interaction site with RNA, whereas Compound 934 shared two interaction sites with RNA, yet there was no overlap of sites between the two compounds (Figures 3C, D). These results suggest that both these compounds may be involved in competitive RNA binding inhibition of PCBP1 through unique inhibitory mechanisms.

To further investigate the ability of Compound 102 and Compound 934 to interfere in the PCBP1–RNA interaction, we deconstructed the crystallographic structure of the PCBP1–RNA complex by individually conducting nucleic acid docking for three scenarios: RNA with PCBP1, RNA with the PCBP1–Compound 102 complex, and RNA with the PCBP1–Compound 934 complex. The PCBP1–compound complexes were generated based on trajectories from the MD simulations. These docking exercises discovered a discernible decrease in docking efficacy between RNA and PCBP1 upon interaction with the compounds, with the effect of Compound 102 on docking being particularly pronounced (Figures 4C–E; Table 1). The RMSD for RNA re-docking onto PCBP1 shows a minor variation of 0.33 Å from the initial co-crystal structure. However, the RMSD increased significantly to 17.84 Å and 15.34 Å when Compound 102 or Compound 934 was introduced, respectively. These findings highlight the substantial impact of these compounds on the PCBP1–RNA interaction.

**TABLE 1:** Docking score and ligand RMSD of PCBP1–RNA complex after introducing compound 102 or compound 934.

	None	Compound 102	Compound 934
Docking score	−234.85	−195.38	−224.34
Confidence score	0.8451	0.7125	0.8156
Ligand RMSD (Å)	0.33	17.84	15.34

Building on our docking results, we undertook a 100 ns MD simulation to further explore how the complexes behave. The comparative analysis of complex conformations before and after the simulation showed that without the addition of the compounds, the RNA segment remained stably anchored within the RNA binding site of PCBP1 (Figures 4F–H). When Compound 102 or Compound 934 was introduced, notable conformational alterations were detected in the RNA binding conformation, including instances where segments of RNA disengaged from the RNA binding site. RNA-protein binding affinity was mainly assessed by Docking Score. Docking Score changed from −234.85 to −195.38 and −224.34 after introduction of Compound 102 and 934, respectively. The difference in Docking Score suggested that both drugs could attenuate the ability of PCBP1 to bind RNA (Table 1). This observation further supports the significant inhibitory influence of the two compounds on the PCBP1–RNA interaction.

R-loops are triple-stranded RNA–DNA hybrids that are formed mainly during transcription, when the DNA non-template chain is replaced by the newly transcribed mRNA (Petermann et al., 2022). To balance R loop accumulation and clearance, RNA processing suppresses R-loop accumulation at all stages of transcription (Petermann et al., 2022). Because the molecular docking and simulation results strongly suggest that Compound 102 and Compound 934 interfere with PCBP1 binding to RNA, we hypothesized that both these compounds impair the ability of PCBP1 to process RNA and lead to intracellular R loop accumulation. Commercial Compound 934 (silychristin, Solarbio, Cat#IS3560) was used for *in vitro* verification and S9.6 antibody was used to detect intracellular R-loop immunofluorescence signals. After treatment with Compound 934 (80 μM, 24 h), we observed significant elevation of S9.6 fluorescence intensity in pancreatic cancer cells (Figures 4I, J), suggesting increased intracellular R-loop accumulation. Together, these findings demonstrate that Compound 102 and Compound 934 interfere with the PCBP1–RNA interaction, thus impairing the ability of PCBP1 to process RNA.

### 3.5 Compound 934 reduces the tolerance towards ROS agent in pancreatic cancer

**FIGURE 5:**
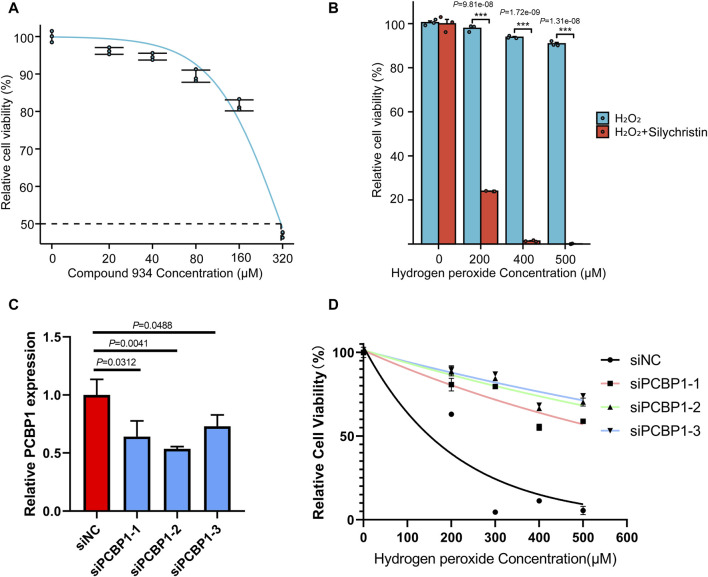
Compound 934 (silychristin) synergize with ROS-modulating agents to promote cell death in pancreatic cancer **(A)** Cell viability measured by CCK-8 assay after treatment of Compound 934 concentration gradients. **(B)** Cell viability measured by CCK-8 assay after 24 h-treatment of hydrogen peroxide concentration gradients alone or accompanied by Compound 934 (silychristin, 160 μM). **(C)** Knock down of PCBP1 by siRNA in PANC-1. PCBP1 expression was measured by RT-qPCR after 48 h transfection. **(D)** Cell viability measured by CCK-8 assay after 24 h-treatment of hydrogen peroxide concentration gradients and Compound 934 (silychristin, 160 μM) in siNC or siPCBP1 PANC-1 cells.

To evaluate the *in vitro* effect of Compound 934, we determined cell viability by CCK-8 assay after 24 h treatment with a Compound 934 concentration gradient (Figure 5A, IC50 = 320 µM). RNA oxidative damage is an early event preceding cell death (Wu and Li, 2008). Considering that PCBP1 exhibits highly selective binding to heavily oxidized RNA via o8G residues located nearby on the RNA strand, interfered from the competitive interaction of Compound 934, we propose that Compound 934 may promote ROS agent-induced cell death in pancreatic cancer. After 24 h treatment with hydrogen peroxide concentration gradients alone or together with Compound 934 (160 µM), we found that Compound 934 significantly enhanced the inhibition effect of RNA agent hydrogen peroxide on pancreatic cancer cell viability (Figure 5B). We knocked down PCBP1 by siRNA in PANC-1 cells (Figure 5C). Then treated the cells with silychristin (160 μM) and hydrogen peroxide concentration gradients. After 24 h treatment cell viability was measured by CCK-8 assay (Figure 5D). We found that PCBP1 knock down did not sensitize PANC-1 cells to hydrogen peroxide when treated together with silychristin, which indicated that synergistic effects between ROS agent and silychristin was indeed through targeting PCBP1. Therefore, targeting of PCBP1 by Compound 934 may synergize with ROS-modulating agents to kill pancreatic cancer cells.

### 3.6 Dynamic simulations between the two compounds and PCBP1 in intracellular and *in vivo* backgrounds

**FIGURE 6:**
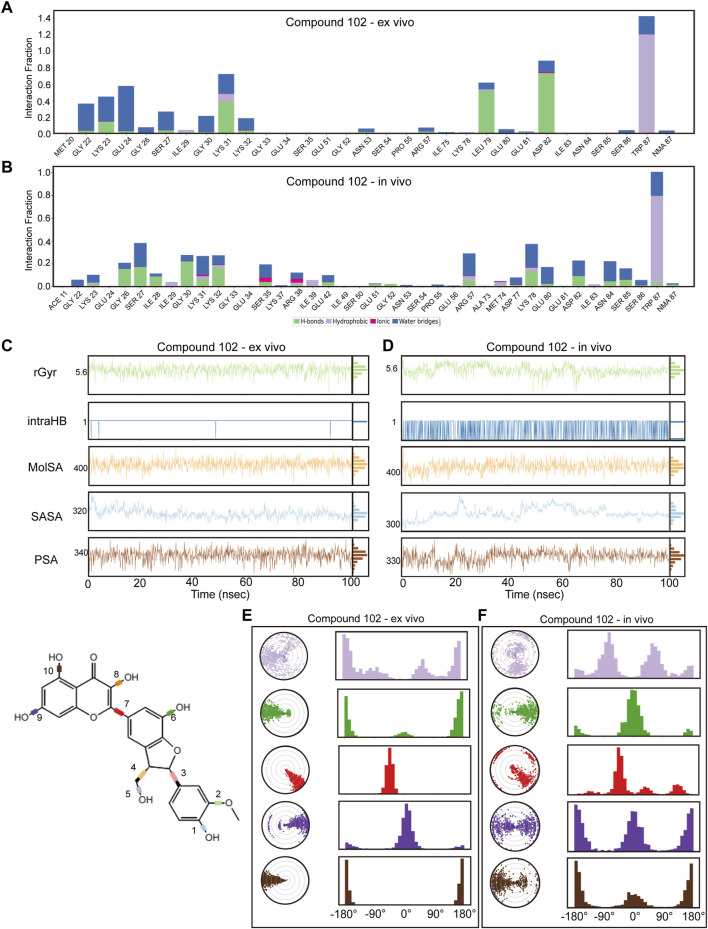
Verification of dynamic simulations of Compound 102 within *ex-vivo* and *in-vivo* settings **(A,B)** Types of interactions between different residues and Compound 102 within *ex-vivo* and *in-vivo* settings. **(C,D)** Radius of Gyration (rGyr), Intramolecular Hydrogen Bonds (intraHB), Molecular Surface Area (MolSA), Solvent Accessible Surface Area (SASA), and Polar Surface Area (PSA) within *ex-vivo* and *in-vivo* settings for Compound 102. **(E,F)** torsion angle radar plots of Compound 102 within different settings.

**FIGURE 7:**
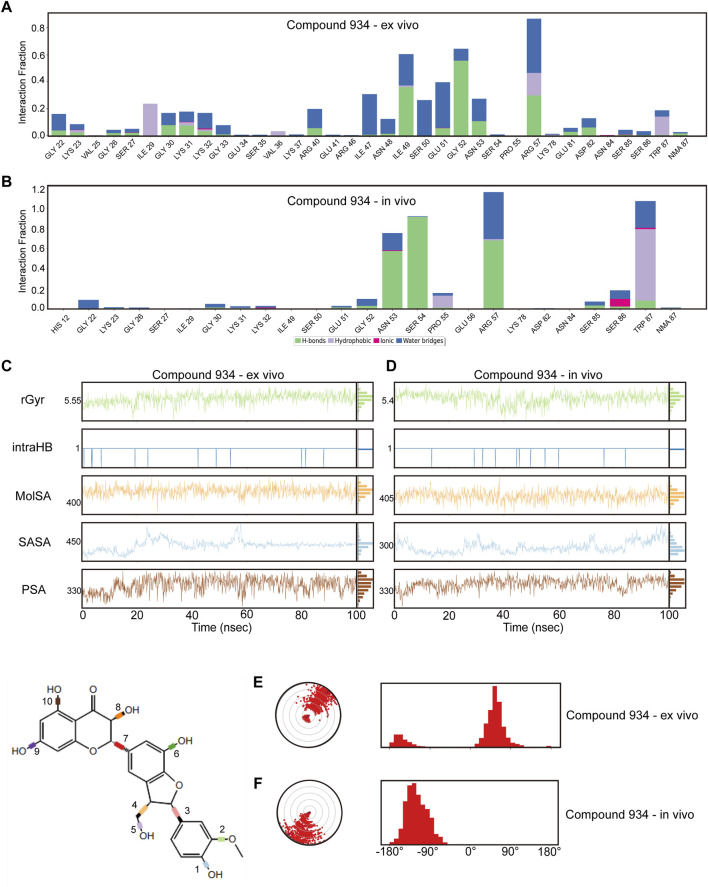
Verification of dynamic simulations of Compound 934 within *ex-vivo* and *in-vivo* settings **(A,B)** Types of interactions between different residues and Compound 934 within *ex-vivo* and *in-vivo* settings. **(C,D)** Radius of Gyration (rGyr), Intramolecular Hydrogen Bonds (intraHB), Molecular Surface Area (MolSA), Solvent Accessible Surface Area (SASA), and Polar Surface Area (PSA) within *ex-vivo* and *in-vivo* settings for Compound 934. **(E,F)** torsion angle radar plots of Compound 934 within different settings.

To guarantee the adaptability of the two compounds in both intracellular and *in vivo* settings, we carried out dynamic simulations for each environment using results from the former *ex vivo* experiments as benchmarks to evaluate potential impacts on the binding efficacy of Compound 102 and Compound 934 under different conditions. For Compound 102, interactions with PCBP1 residues were notably consistent across environments, with a tendency towards conservation in both the residues involved and the mode of interaction (Figures 6A, B; Supplementary Figure S6A). For Compound 934, interactions with low-frequency PCBP1 residues decreased progressively from *ex vivo* to intracellular and subsequently to *in vivo* environments, demonstrating enhanced adaptability (Figures 7A, B; Supplementary Figure S7A).

Further analysis from the perspective of the compounds focused on variations in their radius of gyration, intramolecular hydrogen bonds, molecular surface area, solvent accessible surface area, and polar surface area across different settings. For Compound 102, minimal variations were observed in these dynamic parameters across environments, with a noted increase in intramolecular hydrogen bonds in response to ionic and thermal fluctuations (Figures 6C, D; Supplementary Figure S6B). Compound 934 similarly demonstrated robust environmental adaptability, with negligible variations in the dynamic parameters, underscoring its superior environmental resilience compared with that of Compound 102 (Figures 7C, D; Supplementary Figure S7B).

**FIGURE 8:**
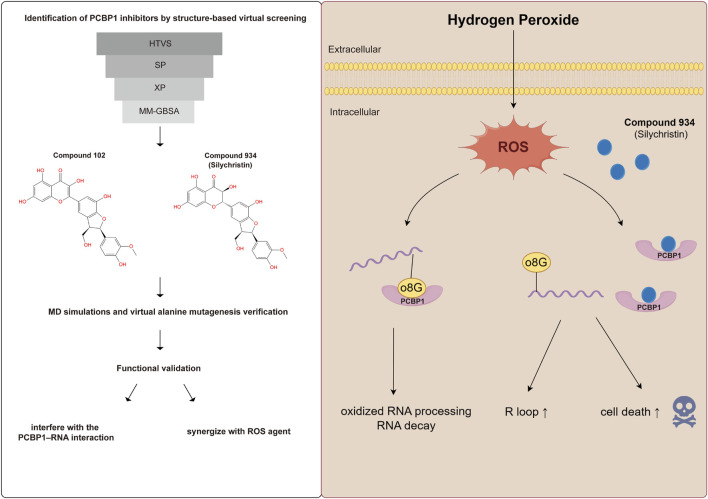
Graphic abstract of this study.

On the structural level, analysis of the two compounds using torsion angle radar plots highlighted significant adaptive changes in the compounds when transitioning between intracellular and *in vivo* environments. For compound 102, notable changes were observed in the torsion angles of bonds 5, 6, 7, 9, and 10, suggesting adaptations to new environmental conditions (Figures 6E, F; Supplementary Figures S6C–E). The stability of Compound 934 was again evident, because only the torsion angle range of bond 7 expanded by approximately 180 degrees (Figures 7E, F; Supplementary Figures S7C–E). This structural analysis accentuates the adaptability of Compound 934 and the nuanced environmental responses of the two compounds; Compound 934 had a more stable and adaptable profile than Compound 102 had in varying physiological conditions. Graphic abstract of this study was showed in Figure 8.

## 4 Discussion

ROS play an important role in inducing PDAC cell proliferation and survival, as well as in initiating hypoxia-dependent epithelial–mesenchymal transition in PDAC (Chang and Pauklin, 2021). Considering the lack of effective targets in PDAC, many drugs directly or indirectly targeting ROS metabolism have been developed, including gemcitabine and erastin, yet significant room remains for improving efficacy (Gorrini et al., 2013). Induced by ROS, o8G is the most abundant among oxidized bases, and its accumulation in mRNA can lead to detrimental protein synthesis (Ishii and Sekiguchi, 2019). Thus, o8G-modulating factors are essential anti-oxidant targets to inhibit the elimination of oxidized RNA and improve the efficacy of existing ROS drugs. Directly written by ROS, o8G can be read by RNA-binding proteins (YB-1, AUF1, PCBP1, and PCBP2) and ribonucleolytic enzymes (PNPase and APE1) (Hahm et al., 2022). Among them, the role of PCBP1 and PCBP2 in binding and processing oxidized RNA has been well-characterized. PCBP1 knockdown was found to promote polyunsaturated fatty acid peroxidation and ferroptosis-induced cell death in head and neck cancer and bladder cancer (Lee et al., 2022; Luo et al., 2023). PCBP2 shares high similarity with PCBP1 in sequence and structure, yet only patients with high PCBP1 expression had worse overall survival in PDAC. Therefore, it is essential to find high-affinity PCBP1 inhibitors without affecting PCBP2 to minimize off-target effects.

Although previous studies on whether PCBP1’s dimerization influence the processing of o8G-modified RNA are limited, we did have taken dimerization into account before virtual screening. Takashi Ishii et al. used 8-oxoG-containing RNA as the probe to search for proteins that bind to heavily oxidized RNA (Ishii et al., 2018). All proteins that attached to the probes were separated by SDS-gel electrophoresis and silver stained. At position of ∼40 kDa they detected strong signals and the major protein was PCBP1. Although it has been reported that PCBP1 can spontaneously form a homodimer and can form a heterodimer with PCBP2 (Yanatori et al., 2020), we did not observe enhancement of the signal at the predicted dimerization site (∼80 kDa). In addition, as the analyzed structure in PDB showed (ID: 1ZTG), it was one subunit in the dimer binding the upstream while one subunit in another dimer binding the downstream of a segment of RNA. It was different from the case that two PCBP1 subunits bound the RNA fragment together after dimerization. Therefore, we hypothesized that blocking one subunit either upstream or downstream of the RNA would inhibit PCBP1.

By applying high-throughput virtual screening, we identified Compound 102 and Compound 934 as high-specificity PCBP1 inhibitors. The two compounds were differentiated by an additional double bond. Both compounds formed bonds with the Gly-30, Glu-51, Gly-52, Asn-53, and Arg-57 residues of PCBP1, and Compound 934 formed additional hydrogen bonds with Lys-23 and Lys-31. MD simulations show that the flexibility of PCBP1 was largely confined by the reduced flexibility in positions 12–18, 28–47, and 60–77, coupled with increased in flexibility in positions 19–27, 48–59, and 78–87. Molecular interaction analysis showed that the interaction sites between Compound 102 and PCBP1 included Leu-79, Asp-82, and Trp-87, whereas those between Compound 934 and PCBP1 included Ile-49 and Arg-57. Because both compounds had strong affinity to PCBP1 and interfere with PCBP1–RNA interaction, we chose the commercial Compound 934 (silychristin) for *in vitro* validation. Although the efficacy of silychristin to suppress pancreatic cancer was low (IC50 = 320 µM), silychristin strongly enhanced ROS agent hydrogen peroxide to induce cell death in pancreatic cancer cells, likely by competitively binding to PCBP1, leading to impaired oxidized RNA elimination. Our docking results and subsequent cheminformatics analysis provided a theoretical basis for drug development and modification in the future.

The correlation between R-loop accumulation and o8G modulator has not been reported previously. R-loops are three-stranded RNA–DNA structures that are formed by annealing of an RNA strand to the non-template chain of DNA (Grunseich et al., 2018), which is known to modulate genomic stability and cell death. The rapid progress in RNA modifications, including m6A or m5C, has shown that epitranscriptional regulators modulate R-loop production or clearance. Zhang et al. found that m6A-modified RNA at double-strand break sites was stabilized by YTHDC1 and increased the accumulation of R-loops (Zhang et al., 2020). Yang et al. showed that the Fragile X Messenger Ribonucleoprotein (FMRP) was responsible for m5C removal by interacting with m5C eraser TET1, which is highly correlated with R-loop dissolution (Yang et al., 2022). By applying virtual docking and computational MD simulations, we showed that Compound 934 and Compound 102 interfered with the PCBP1–RNA interaction and impair the ability of PCBP1 to process RNA, leading to intracellular R loop accumulation. Our findings suggest that o8G, the most abundant modification in oxidized RNA, may correlate with intracellular R-loop homeostasis when cells are faced with oxidative stress.

In conclusion, we identified PCBP1 as a potential oncogene in pancreatic cancer. Structure-based virtual screening and computational MD simulations screened out Compound 102 and 934 as small molecule inhibitors that specifically target the RNA binding domain of PCBP1, but not that of PCBP2. Both compounds interfere with the PCBP1–RNA interaction, thus impairing the ability of PCBP1 to process RNA, leading to R loop accumulation. Compound 934 (silychristin) synergizes with ROS agent to strongly improve hydrogen peroxide-induced cell death in pancreatic cancer cells. Our findings provide a theoretical basis for development and modification of drugs targeting PCBP1, which showed promising synergistic effects with ROS-modulating drugs in pancreatic cancer.

## Data Availability

The original contributions presented in the study are included in the article/Supplementary Material, further inquiries can be directed to the corresponding authors.

## Abbreviations

PCBP1- poly (rC)-binding protein 1; ROS - reactive oxygen species; o8G - 8-oxoguanine; RBP - RNA binding protein; ADME - adsorption, distribution, metabolism and excretion; PCBP2 - poly (rC)-binding protein 2; RMSD - Root Mean Square Deviation; RMSF - Root Mean Square Fluctuation; rGyr - Radius of Gyration; intraHB - Intramolecular Hydrogen Bonds; MolSA - Molecular Surface Area; SASA - Solvent Accessible Surface Area; PSA - Polar Surface Area; EMT - epithelial-to-mesenchymal transition; PDAC - Pancreatic ductal adenocarcinoma; HTVS - high-throughput virtual screening; hnRNP - heterogeneous nuclear ribonucleoprotein.
