# Supplementary material for: Discovery of an 8-oxoguanine regulator PCBP1 inhibitor by virtual screening and its synergistic effects with ROS-modulating agents in pancreatic cancer
Source: Front Mol Biosci. 2024 Aug 7;11:1441550. doi: 10.3389/fmolb.2024.1441550 (PMC11336162; doi:10.3389/fmolb.2024.1441550)
Supplement: Supplementary file 4 [file Image1.pdf]

Supplementary Figures

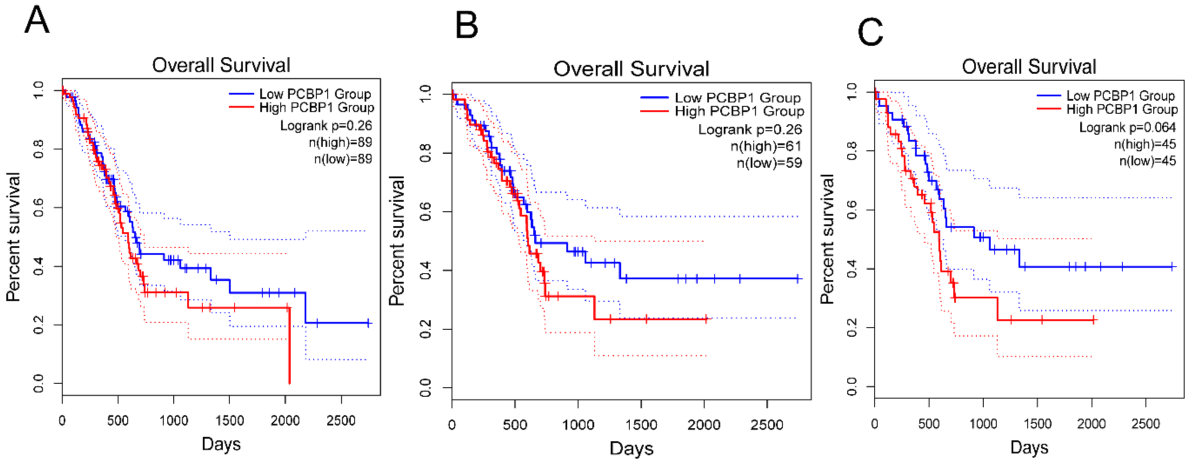

Supplementary Figure 1 Overall survival of different PCBP1 expression thresholds in TCGA-PAAD

A. Overall survival of high/low expression based on dichotomy

B. Overall survival of high/low expression based on tertile

C. Overall survival of high/low expression based on quartile

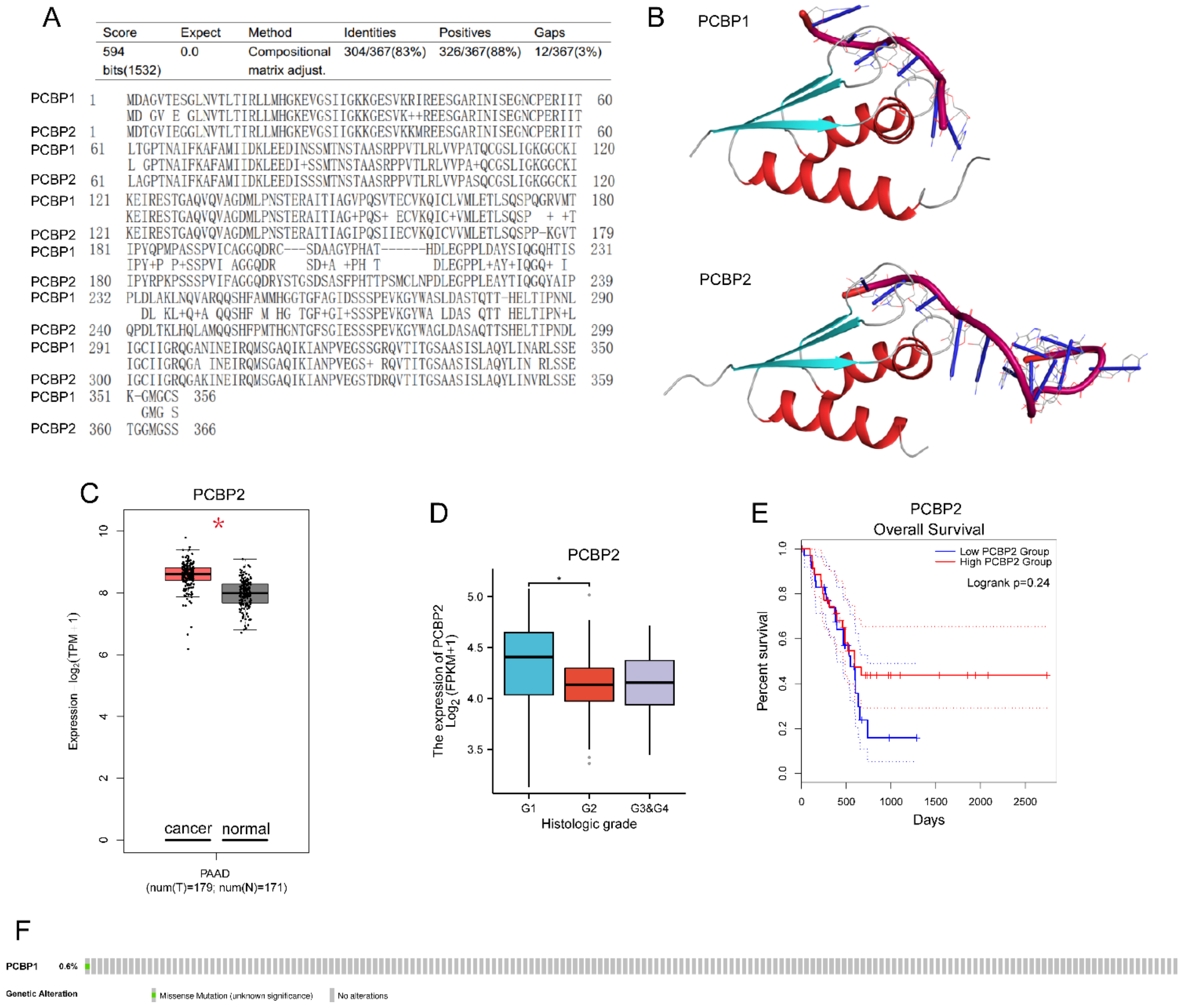

Supplementary Figure 2 PCBP2 shares high homology with PCBP1 yet not correlates with poor prognosis in pancreatic cancer and PCBP1 mutants in TCGA-PAAD

(A) Sequence comparison of PCBP1 and PCBP2 from BLASTP (B) 3D structure of PCBP1 and PCBP2 from PDB database (Entry ID: 3VKE; 2PY9) (C) Expression of PCBP2 in pancreatic cancer tissues and normal tissues in TCGA and GTEx (cancer vs normal, $P = 2.38e\text{-}30$). (D) Expression of PCBP2 in different histological grading stage group of TCGA-PAAD (G1 vs G2, $P = 0.0491$). (E) Overall survival of low/high expression of PCBP2, 20% and 80% are cut-off value for low or high expression group respectively. (F) Oncoprint of PCBP1 mutants in TCGA-PAAD.

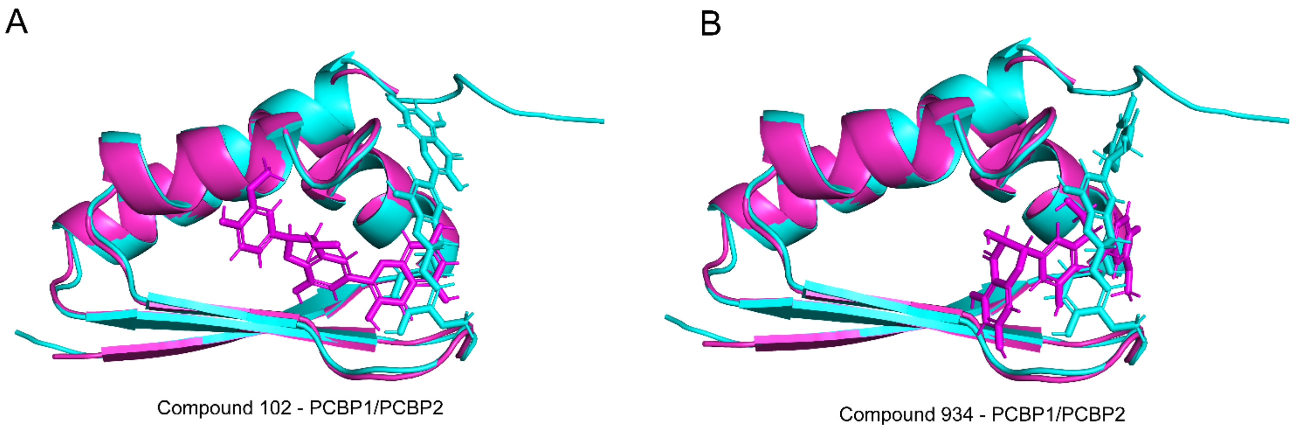

Supplementary Figure 3 Molecular docking of Compound 102/934 binding to PCBP1/PCBP2

(A) molecular docking of Compound 102 binding to PCBP1 or PCBP2. Compound 102-PCBP1 complex colored in magenta; Compound 102-PCBP2 complex colored in blue;

(B) molecular docking of Compound 934 binding to PCBP1 or PCBP2. Compound 934-PCBP1 complex colored in magenta; Compound 934-PCBP2 complex colored in blue;

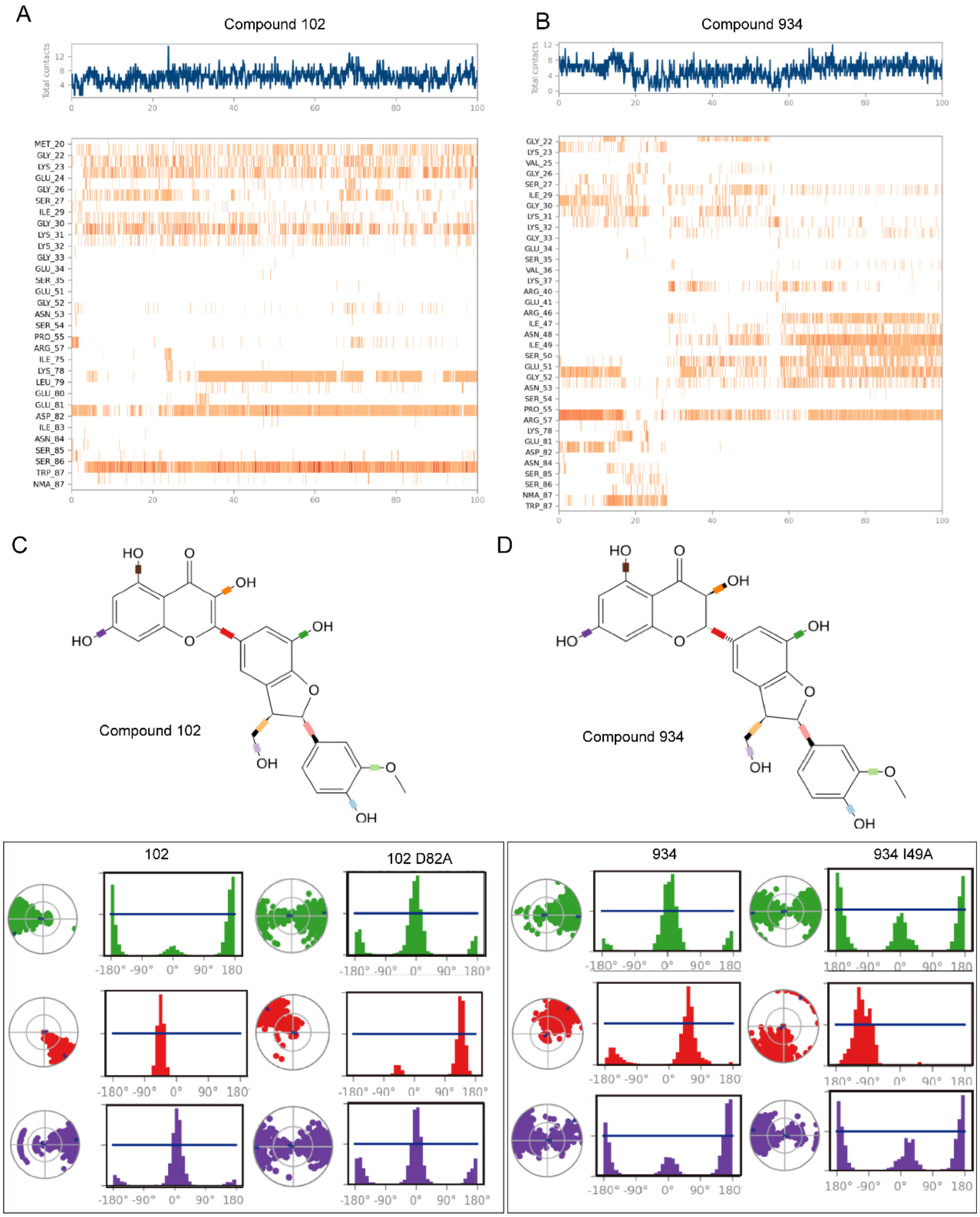

Supplementary Figure 4 Molecular dynamic simulations of interaction between compounds and PCBP1

(A-B) Dynamic changes of Compound 102 and 934’s contacts with PCBP1 residues over time (C-D) Torsion angle radar plots of Compounds Fit on wild-type or mutant PCBP1 Complexes

A

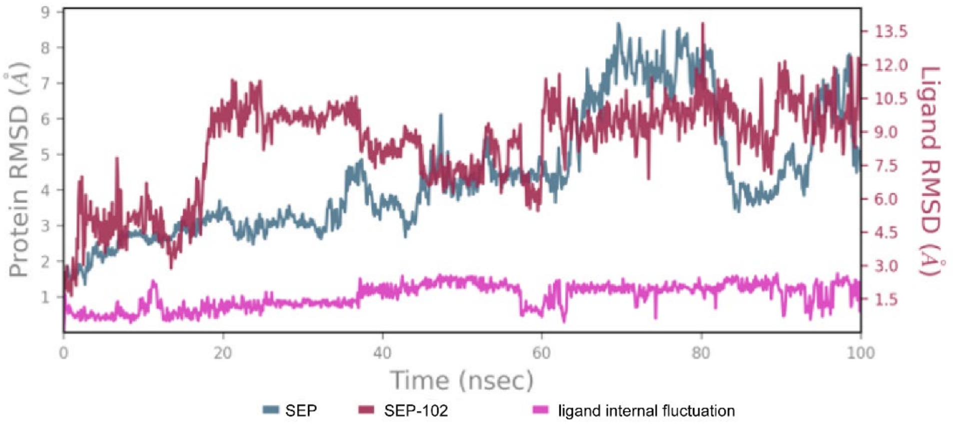

B

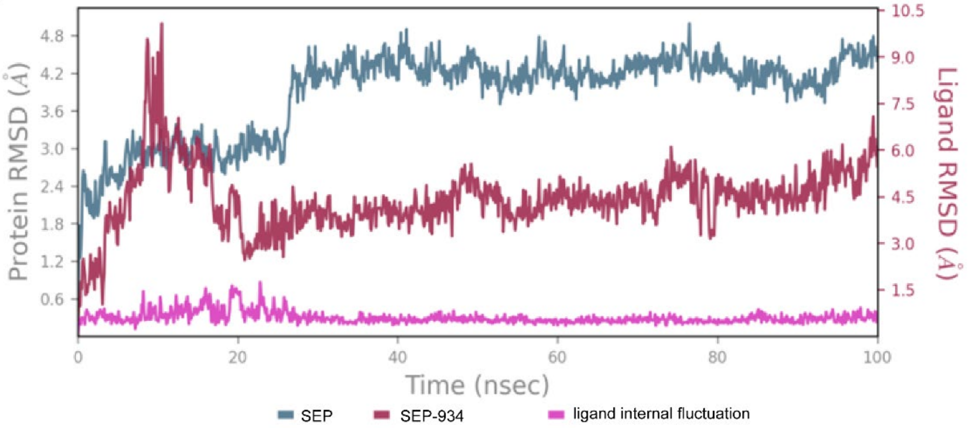

Supplementary Figure 5 RMSD for Compound 102/934 and phosphorylated PCBP1 (Ser43)

A. Dynamic changes of RMSD for Compound 102 and phosphorylated PCBP1 (Ser43) over time

B. Dynamic changes of RMSD for Compound 934 and phosphorylated PCBP1 (Ser43) over time

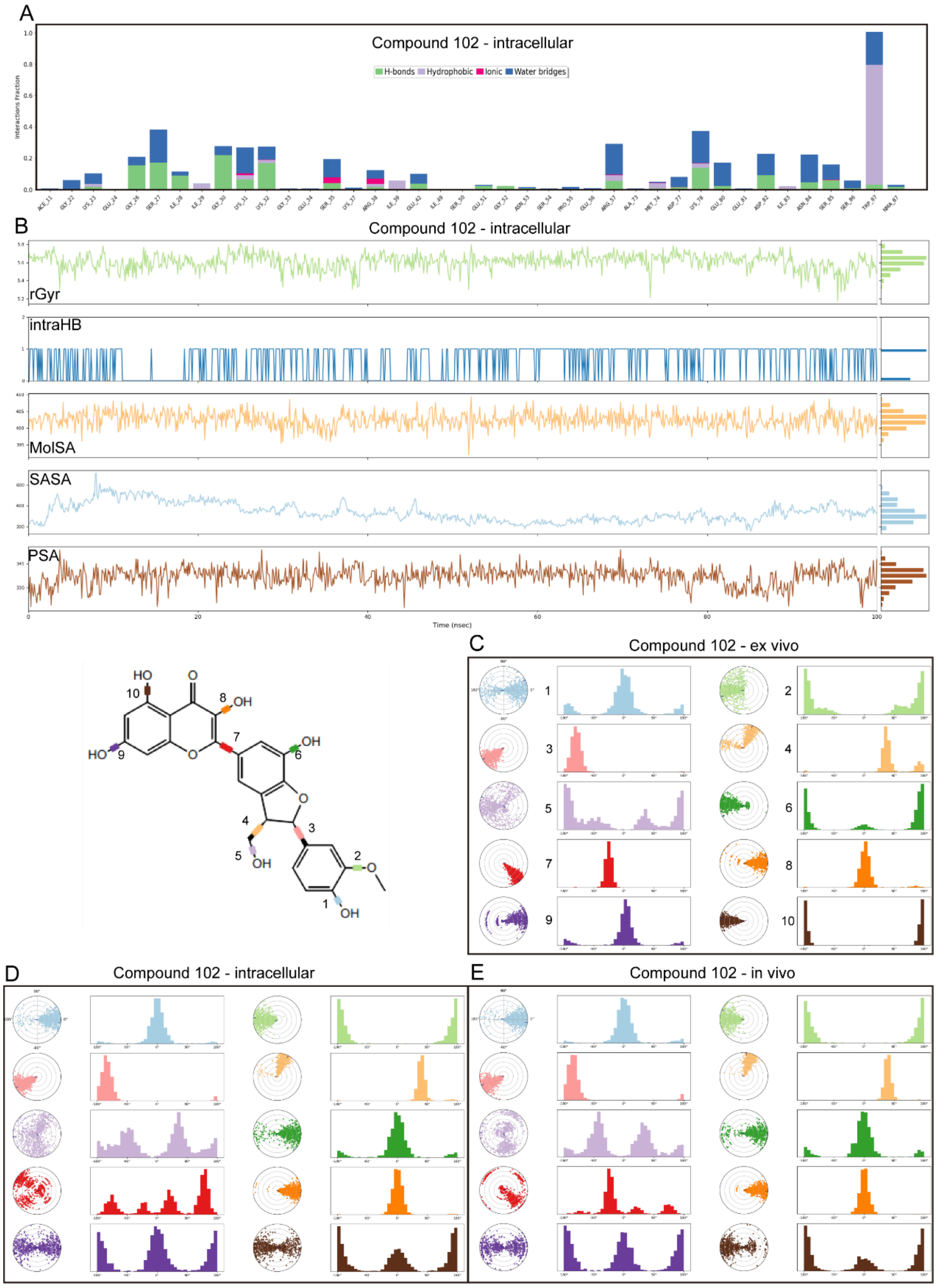

Supplementary Figure 6 Dynamic simulations of 102 within different settings

(A) Types of interactions between different residues and Compound 102 intracellular. (B) Radius of Gyration (rGyr), Intramolecular Hydrogen Bonds (intraHB), Molecular Surface Area (MolSA),

Solvent Accessible Surface Area (SASA), and Polar Surface Area (PSA) of 102 within intracellular settings (C-E) torsion angle radar plots of 102 within different settings

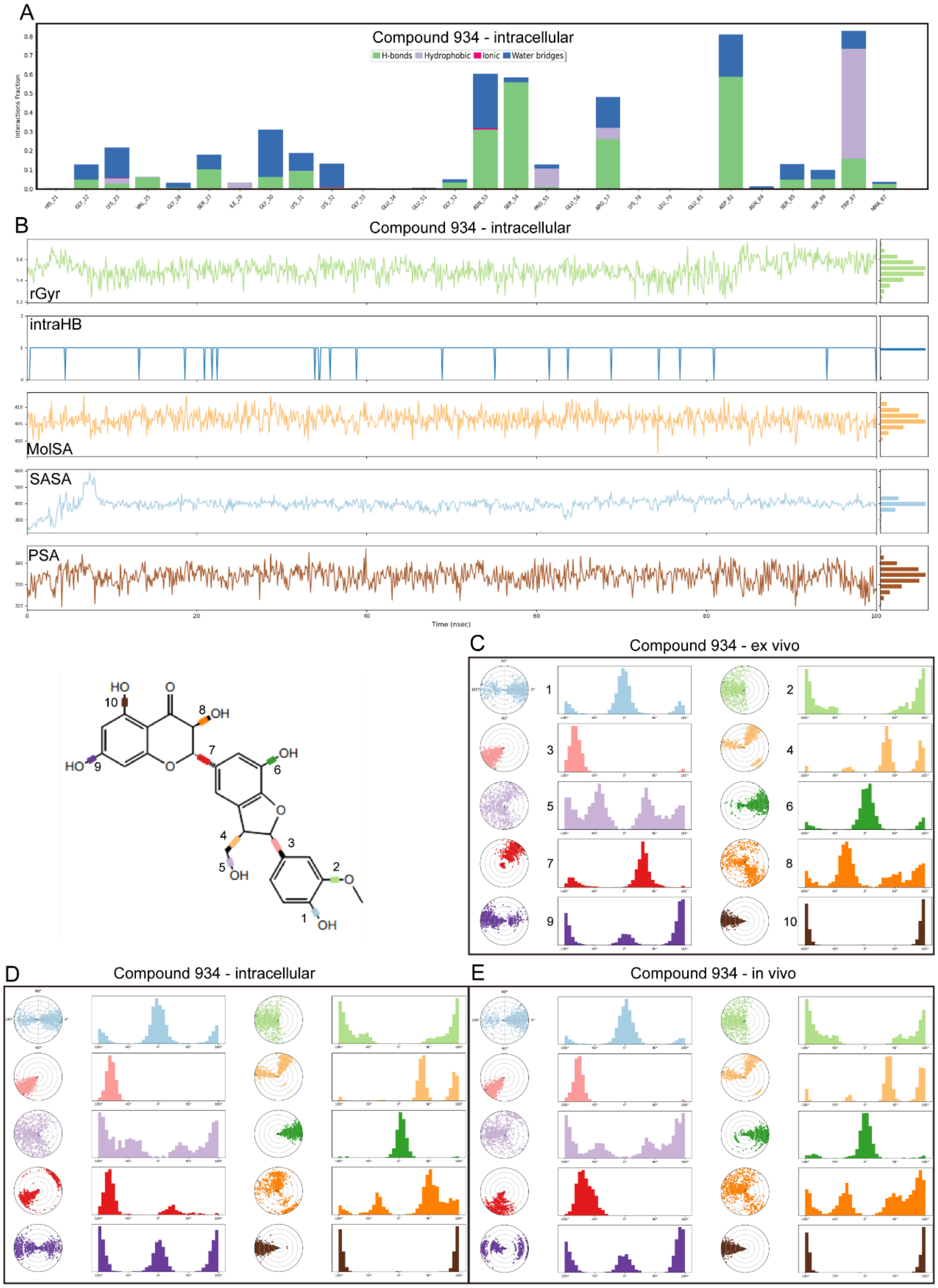

Supplementary Figure 7 Dynamic simulations of 934 within different settings

(A)Types of interactions between different residues and Compound 934 intracellular. (B) Radius of Gyration (rGyr), Intramolecular Hydrogen Bonds (intraHB), Molecular Surface Area (MolSA),

Solvent Accessible Surface Area (SASA), and Polar Surface Area (PSA) of 934 within intracellular settings (C-E) torsion angle radar plots of 934 within different settings
